# Monitoring bacterial contamination of blood components at the Croatian Institute of Transfusion Medicine—Evolution of strategies and results in a 14‐year period (2011–2024)

**DOI:** 10.1111/vox.70175

**Published:** 2026-01-04

**Authors:** Ivanka Batarilo, Mia Slade‐Vitkovic, Lidija Rukavina, Jadranka Gulan Harcet, Julijana Ljubicic, Adrijana Grdic, Marko Karlo Radovcic, Matea Vinkovic, Irena Jukic, Tomislav Vuk

**Affiliations:** ^1^ Microbiology Department Croatian Institute of Transfusion Medicine Zagreb Croatia; ^2^ Quality Control Department Croatian Institute of Transfusion Medicine Zagreb Croatia; ^3^ Quality Assurance Department Croatian Institute of Transfusion Medicine Zagreb Croatia; ^4^ Blood Component Department Croatian Institute of Transfusion Medicine Zagreb Croatia; ^5^ Internal Medicine Department University of Zagreb School of Medicine Zagreb Croatia; ^6^ Medical Division Croatian Institute of Transfusion Medicine Zagreb Croatia; ^7^ Quality Management Division Croatian Institute of Transfusion Medicine Zagreb Croatia

**Keywords:** bacterial contamination, bacterial screening, blood components

## Abstract

**Background and Objectives:**

This study presents the results and experiences of bacterial testing of blood components (BCs) at the Croatian Institute of Transfusion Medicine during the period 2011–2024.

**Materials and Methods:**

During the 14‐year period, 74,283 BCs were tested. Among these, 20,231 components (8345 red blood cell concentrates, 5729 platelet concentrates [PCs] and 6157 plasma units) were tested as part of statistical quality control (QC). In addition, 100% bacterial screening was implemented for aphaeresis platelets in November 2019 and for pooled platelets in October 2022 with 17,187 aphaeresis platelets and 36,865 pooled platelets tested by the end of 2024. All pooled platelets were tested using the large‐volume delayed sampling (LVDS) method, whereas 9596 aphaeresis platelets were tested using the two‐step method (from November 2019 to November 2022) and 7591 using LVDS (from November 2022 to December 2024). BCs were sampled and inoculated into both aerobic and anaerobic culture bottles and incubated at 36 ± 1°C for 7 days.

**Results:**

As part of the statistical QC, 20,231 BCs (5729 PCs) were tested, resulting in a confirmed contamination rate of 0.09% (0.14% for PCs). Since the implementation of universal screening, 54,052 PCs have been examined, with a confirmed positivity rate of 0.18%. The most frequently detected organism was *Cutibacterium acnes*.

**Conclusion:**

The confirmed positive rate of bacterial testing in our study and the isolates from positive cultures are comparable to similar studies. Active bacterial screening of BCs, among other measures, remains a critical step for preventing transfusion‐associated bacterial infections.

**Highlights**

- Active bacterial screening of blood components (BCs) is an important safety measure reducing risks of transfusion‐transmitted bacterial infection.
- The incidence of bacterial contamination at the Croatian Institute of Transfusion Medicine was found to be consistent with that reported in the previous literature.
- Skin commensals (primarily *Cutibacterium acnes*) are the most frequently detected bacteria in contaminated BCs.

## INTRODUCTION

While blood components (BCs) are irreplaceable in the treatment of many medical conditions, their use carries risks. According to Food and Drug Administration (FDA) data over the years, microbial transmission ranks third and fifth among the most common causes of reported transfusion‐related fatalities in the United States [1, 2]. Moreover, symptoms of transfusion‐associated bacterial sepsis can be easily overlooked and underreported even in high‐income countries [3, 4, 5, 6, 7, 8].

The origin of bacterial contamination of BCs can be exogenous or result from endogenous sources. Most microorganisms originate from the skin, including *Staphylococcus*, *Corynebacterium* and *Propionibacterium* species [5, 7, 8]. Low counts of bacteria introduced during the venipuncture may go undetected but can proliferate to life‐threatening levels by the time of transfusion. While Gram‐positive pathogens are more common, contamination with Gram‐negative bacteria is more likely to result in septic fatalities [9].

The contamination rate of platelet concentrates (PCs), the risk of sepsis and the incidence of fatal septic reactions are higher than with other BCs. The detection rate of contaminated platelets increases with storage time due to bacterial growth to detectable levels. Although PCs contamination occurs at an approximately one log higher frequency, red cell concentrates (RCCs) are transfused more frequently and should not be overlooked in terms of infectious risk [10].

Active screening for bacterial contamination of BCs is an important measure in preventing transfusion‐transmitted infection (TTI), including culture‐based and rapid detection tests [7, 8, 11]. There is currently no consensus on the optimal timing of sampling, leading to variation in detection sensitivity [12]. To enhance the safety of platelet transfusions, the FDA's updated guidelines recommend single‐step and two‐step bacterial risk control strategies [13, 14]. Single‐step strategies include large‐volume delayed sampling (LVDS), defined as a culture sampled at least 36 h after collection with at least 16 mL of product, and/or pathogen reduction technologies (PRTs). Two‐step strategies involve both primary and secondary testing during the platelet shelf life. Implementation of the LVDS algorithm has led to enhanced component safety and a threefold reduction in the incidence of septic transfusion reactions [6, 12, 15].

The first results and experiences of bacterial testing of BCs at the Croatian Institute of Transfusion Medicine (CITM) were published in 2012 for the period 2000–2010 [16]. Here we report the results of all BC bacterial testing programmes carried out at the CITM Department of Microbiology during the period 2011–2024.

## MATERIALS AND METHODS

### Bacterial testing

During the study period from 2011 to 2024, a total of 20,231 BCs were screened for bacterial contamination as part of statistical quality control (QC) procedures, in accordance with the recommendations of the Paul‐Ehrlich Institute [17]. The tested components included 8345 RCCs, 5729 PCs and 6157 plasma products (Table 1). Between November 2019 and November 2022, all aphaeresis‐derived PCs (*n* = 9596) were screened for bacterial contamination using a two‐step detection method. In October 2022, a LVDS strategy was implemented for pooled PCs and in November 2022 for aphaeresis PCs. By the end of 2024, a total of 36,865 pooled PCs (each prepared from four buffy coats) and 7591 aphaeresis PCs were examined for bacterial contamination using the LVDS approach (Table 2). Following the implementation of microbiological screening, the shelf life of PCs has been extended from 5 to 7 days. All BCs were sampled with the MacoPharma sampling bags (MacoPharma, France).

**TABLE 1:** Contamination of blood components tested as part of statistical control in the period 2011–2024.

Blood component	Tested (*n*)	IP		CP		UP		FP	
		*n*	%	*n*	%	*n*	%	*n*	%
RCCs	8345	26	0.31	8	0.10	1	0.01	17	0.20
PCs	5729	29	0.51	8	0.14	1	0.02	20	0.35
Plasma	6157	6	0.10	2	0.03	1	0.02	3	0.05
Total	20,231	61	0.30	18	0.09	3	0.01	40	0.20

Abbreviations: CP, confirmed positive; FP, false‐positive; IP, initially positive; *n*, number; PCs, platelet concentrates; RCCs, red cell concentrates; UP, unconfirmed positive.

**TABLE 2:** Comparison of platelet component contamination after implementation of 100% screening in the period November 2019–2024.

Blood component	Tested (*n*)	IP		CP		UP		FP	
		*n*	%	*n*	%	*n*	%	*n*	%
Aphaeresis PCs ‘two step’	9596	36	0.38	14^a^	0.15	7	0.06	16	0.17
Aphaeresis PCs LVDS	7591	33	0.43	10	0.13	5	0.07	18	0.24
Pooled PCs	36,865	159	0.43	73	0.20	36	0.10	50	0.14
Total	54,052	228	0.42	97	0.18	48	0.09	84	0.16

Abbreviations: Aphaeresis PCs LVDS, aphaeresis platelet concentrates screened using large‐volume delayed sampling; Aphaeresis PCs ‘two step’, aphaeresis platelet concentrates screened using the ‘two‐step’ method; CP, confirmed positive; FP, false‐positive; IP, initially positive; *n*, number; UP, unconfirmed positive.

^a^ One confirmed positive PC was not initially positive. *Staphylococcus aureus* was found during secondary cultivation on day 5 and later confirmed in the retention sample.

Bottles with nutrient media for aerobic (BPA) and anaerobic (BPN) cultivation from BioMérieux, France, were used for the inoculation of BCs from QC, as well as for screening of PCs using two‐step and LVDS methods. Approximately 20 mL of BCs were collected into sampling blood bags. A minimum of 8 mL was inoculated into each BPA and BPN bottle applying aseptic methods in a laminar flow cabinet. Both bottles were placed in the BacT/ALERT 3D® and BacT/ALERT Virtuo® (BioMérieux, France) for 7 days at 36 ± 1°C. For microbial identification, after subcultivation of positive bottle samples, Gram staining and manual microorganism identification test kits (API, BioMérieux, France) were used.

During the application of the two‐step method, all aphaeresis PCs on stock were subjected to secondary cultivation on day 5. A volume of 100 μL of the BC was inoculated onto two Columbia agar plates and incubated under aerobic and anaerobic conditions for 24 and 48 h, respectively. At the same time, all unused and expired PCs on day 7 were rescreened following the initial screening protocol; a minimum of 8 mL of the PC was inoculated into both aerobic and anaerobic culture bottles and incubated for 7 days using BacT/ALERT system.

As of November 2022, LVDS strategy was introduced. Due to increased workload, a new device (BacT/ALERT® Virtuo®) was implemented in March 2023. All PCs, whether prepared from whole blood donations or collected via aphaeresis from a single donor, were tested 36–48 h after donation to allow bacterial proliferation to detectable levels [18]. Samples of 8–10 mL were inoculated into both BPA and BPN bottles and incubated in the BacT/ALERT system for 7 days at 36 ± 1°C. All PCs were incubated for 16 h before being released with a ‘negative to date’ result. After 7 days of incubation, a final negative result was reported. If the system reported a positive initial signal, the implicated BC and all associated components were either recalled or quarantined. In cases where a BC had already been transfused, the hospital was immediately notified of a potential contamination of the transfused unit.

### Prevention of laboratory contamination of BCs

Laboratory contamination of BCs during microbiological testing is prevented by working in a protective Class A microbiology safety cabinet located in a restricted‐access area, disinfected prior to use. Staff wear disposable protective clothing, use disinfected equipment and clean the cabinet after each session. Environmental microbiological monitoring was performed using a combination of methods including exposure plates for air quality, surface contact plates and glove prints to assess microbial burden during each work session.

### Culturing and identification

All samples identified as positive by BacT/ALERT were aseptically processed to identify possible microorganisms. Using a sterile syringe, a 10‐mL sample from the bottle with the positive signal was collected and centrifuged. The supernatant was decanted, and 10 μL of the precipitate was spread with a sterile loop to two Columbia agar plates, one incubated under aerobic and the other under anaerobic conditions at 36 ± 1°C for 24 and 48 h, respectively. A Gram stain was prepared from the precipitate on a sterile slide. A negative bottle from the aerobic/anaerobic pair of the same sample was incubated for up to 14 days. All BCs related to the initially positive (IP) sample were recalled. Those that were not transfused were sampled and subjected to bacterial screening. For microbial identification, Gram staining, morphology of grown colonies and manual microorganism identification test kits (API) were used. If the smear was negative, a new sample was withdrawn from the bottle, transferred into a new BPA or BPN bottle and incubated for another 7 days.

All IP results were later determined to be confirmed positive (CP), unconfirmed positive (UP) or false‐positive (FP) results, depending on the bacterial contamination of associated BCs. For each IP PC, a retention sample of the PC, if available, is sent for bacterial screening along with all available associated BCs (samples of RCCs and plasma). If the same microorganism was found in the retention sample and/or any associated BC, the result was considered CP. If the microorganism was not confirmed in any of the related BCs or in the retention sample, the result was considered indeterminate or UP. In FP BCs, no microorganism was isolated after a positive signal from BacT/ALERT.

Ethical approval was not required for this study as it was based solely on the analysis of routinely collected data from bacterial testing of BCs, and no identifiable donor or patient data were used.

### Statistical analysis

Statistical analysis was performed using Microsoft Excel. To determine statistical significance between observed groups of BCs, the *χ* ^2^‐test was used. A *p*‐value ≤0.05 was considered statistically significant.

## RESULTS

BCs prepared at CITM were divided into three large groups: RCCs, PCs and plasma products (fresh frozen plasma and cryoprecipitate).

During the 14‐year period, a total of 74,283 BCs were examined. Of these, 20,231 BCs were tested as part of QC between 2011 and 2024, and 54,052 PCs were screened using a 100% screening method (Tables 1 and 2).

As part of statistical control, 8345 RCCs, 5729 PCs and 6157 plasma products were tested (Table 1). There were 61 IP results, out of which 18 were CP (0.09%), 3 UP (0.01%) and 40 FP (0.20%). Confirmed bacterial contamination was mostly recorded in PCs (0.14%, *n* = 8).

Table 2 shows a comparison of bacterial contamination rates of the two types of PCs (obtained by aphaeresis and from whole blood) after the 100% microbial screening was implemented. Since November 2019, when universal screening of aphaeresis PCs was introduced, a total of 17,187 aphaeresis PCs have been tested (9596 were screened using the two‐step method and 7591 using the LVDS method). During the period from October 2022 to 2024, 36,865 pooled PCs (obtained from four buffy coats each) were screened, which accounts for 147,460 donations and represents 76.4% of all collected blood donations (donations whose buffy coats were used in the preparation of pooled PCs).

Bacterial contamination was confirmed in 0.14% (*n* = 24) of all aphaeresis PCs since November 2019. Bacterial contamination of all PCs obtained from whole blood donations since October 2022 was confirmed in 0.20% (*n* = 73). There was no statistically significant difference between the contamination rate of platelets collected by aphaeresis and platelets obtained from whole blood donations (*p* = 0.3). Furthermore, no statistically significant difference was found between contamination rates before and after 100% screening implementation (*p* = 0.5).

### Bacterial isolates

All the isolates identified in BCs were members of the human microbiota, either skin commensals or members of the intestinal flora. Most of the isolates were Gram‐positive bacteria, but Gram‐negative bacilli, such as *Escherichia coli* and *Enterobacter cloacae*, were also detected (Tables 3 and 4). The most common bacterial isolate detected in BCs prepared at CITM was *Cutibacterium acnes*. It was present in all CP RCCs and was the most common in both CP and UP PCs. *C. acnes* is an anaerobe, and accordingly, the largest number of positive bottles were anaerobic. The longest average detection time was recorded for *C. acnes*, 4.3 days.

**TABLE 3:** Bacteria isolated from positive blood components, detection time and culture bottle type, statistical control.^a^

BC	Isolate	CP (*n*)	CP				UP (*n*)	UP			
			BPN (*n*)	Time (h)	BPA (*n*)	Time (h)		BPN (*n*)	Time (h)	BPA (*n*)	Time (h)
PCs	*Cutibacterium acnes*	5	5	104.9							
	*Staphylococcus aureus*	1	1	3.8	1	3.8					
	*Staphylococcus epidermidis*	1	1	2.3	1	2.3					
	*Enterobacter cloacae*	1	1	3.8	1	3.8					
	*Bacillus* spp.						1			1	16.2
RCCs	*C. acnes*	8	8	95.4			1	1	130.5		
Plasma	*C. acnes*	2	2	140.2							
	CoNS						1	1	15.8		

Abbreviations: BC, blood component; BPA, bottle for aerobic cultivation; BPN, bottle for anaerobic cultivation; CoNS, coagulase‐negative staphylococci; CP, confirmed positive; *n*, number; PCs, platelet concentrates; RCCs, red cell concentrates; UP, unconfirmed positive.

^a^ Results of bacterial isolates from PCs after introduction of 100% screening are presented separately (Table 4).

**TABLE 4:** Bacteria isolated from positive platelet components, average detection time and culture bottle type after implementation of 100% screening.

BC	Isolate	CP (*n*)	CP				UP (*n*)	UP			
			BPN (*n*)	Time (h)	BPA (*n*)	Time (h)		BPN (*n*)	Time (h)	BPA (*n*)	Time (h)
Aphaeresis PCs	*Cutibacterium acnes*	16	16	84.7	4	176.1	11	11	101.5	3	191.1
	CoNS	1	1	10.8	1	9.9					
	*Staphylococcus epidermidis*	1			1	14.4					
	*Staphylococcus aureus* ^a^	1	1	3.7	1	3.7					
	*Streptococcus pyogenes*	1	1	24.7	1	15.9					
	*Escherichia coli*	1	1	2.6	1	2.4					
	*Bacillus* spp.	1			1	23.3					
	*Staphylococcus saccharolyticus*	2	2	71.9			1	1	62.3		
Pooled PCs^b^	*C. acnes*	59	59	103.2	15	189.5	26	26	105.7	5	237.6
	*Cutibacterium granulosum*	1	1	94.8			3	3	114.7		
	*S. aureus*	1	1	11.8	1	10.3					
	*S. epidermidis*	5	4	14.6	5	14.3	1	1	28.5		
	*Staphylococcus capitis*						2			2	25.2
	CoNS						1			1	16.4
	Peptostreptococcus group						1	1	39.3		
	*S. saccharolyticus*	7	7	54.5	2	86.0	3	3	90.5		

Abbreviations: BC, blood component; BPA, bottle for aerobic cultivation; BPN, bottle for anaerobic cultivation; CoNS, coagulase‐negative staphylococci; CP, confirmed positive; *n*, number; PCs, platelet concentrates; UP, unconfirmed positive.

^a^ *Staphylococcus aureus* was detected in one aphaeresis PC during secondary cultivation.

^b^ Two bacterial species (*C. granulosum* and CoNS) were isolated from one UP PC pool.

In general, Gram‐negative bacteria were detected rapidly, *E. coli* after 2.4 h and *E. cloacae* 3.8 h of incubation in aerobic bottles. The time required for detection of *Staphylococcus aureus* was like or slightly longer than for Gram‐negative bacteria (range 3.7–10.3 h). Coagulase‐negative staphylococci required slightly longer incubation times, in the range 9.9–28.5 h and *Bacillus* spp. even longer (range 16.2–23.3 h) (Tables 3 and 4).

In our study, the longest detection times for *C. acnes* was 297.5 h (more than 12 days) in pooled PC and 218.6 h in aphaeresis PC, both in aerobic bottles during prolonged cultivation. In these samples, a positive signal was detected earlier in anaerobic bottles, 101.5 and 76.2 h, respectively. Average detection time for *C. acnes* in pooled PCs was 189.5 h, and 176.1 in aphaeresis PCs. Most CP and UP BCs were detected in anaerobic culture bottles, as shown in Table 5. This was expected, since anaerobic bacteria were mostly isolated.

**TABLE 5:** Comparison of positive culture bottles among confirmed positive blood components and unconfirmed positive blood components, statistical control.

Positive bottle	CP			UP		
	PCs	RCCs	Plasma	PCs	RCCs	Plasma
BPN (%)	66.67	100	100	72.73	100	66.67
BPA (%)	3.81	0	0	9.09	0	33.33
BPN + BPA (%)	29.52	0	0	18.18	0	0

Abbreviations: BPA, bottle for aerobic cultivation; BPN, bottle for anaerobic cultivation; CP, confirmed positive; PCs, platelet concentrates; RCCs, red cell concentrates; UP, unconfirmed positive.

### FP results

During the study period, statistical QC testing revealed 40 (0.20%) FP results among all tested BCs, including 20 (0.35%) in PCs (Table 1). The highest number of FP results (84) were detected during the implementation of 100% PC screening (Table 2). The increase in positive results was associated with faster loading of a higher number of culture bottles. To solve this problem, a new Virtuo device was implemented in July 2023. More FP signals were observed in aerobic vials than in anaerobic vials (Table 6). FP signals within 24 h occurred in 36.8% of anaerobic (14/38) and 91.2% of aerobic bottles (83/91).

**TABLE 6:** Frequency of false‐positive results in blood component testing using BacT/ALERT.

BC	BPN		Time (h)	BPA		Time (h)
	*n*	%		*n*	%	
RCCs^a^	4	0.04	2.1	14	0.15	12.1
PCs^b^	32	0.05	25.7	76	0.13	14.1
Plasma	2	0.03	2.1	1	0.01	2.1
Total	38	0.05		91	0.12	

Abbreviations: BC, blood component; BPA, bottle for aerobic cultivation; BPN, bottle for anaerobic cultivation; *n*, number; PCs, platelet concentrates; RCCs, red cell concentrates.

^a^ One RCC was positive in both BPA and BPN.

^b^ Four PCs were positive in both BPA and BPN.

## DISCUSSION

Transfusion‐associated bacterial infections are rare but may be a serious complication that can lead to bacterial sepsis and death. While significant efforts have been made to minimize the risk of transmission, the risk has not been eliminated.

The most common bacterial isolates found in contaminated BCs belong to the human skin flora, such as staphylococci, corynebacteria and bacillary species as well as anaerobic bacilli [19]. The most common bacteria isolated from BCs in our study was *C. acnes*. It was the most common isolate from all three large groups of products (RCCs, PCs and plasma products), and it was the only bacterium isolated from CP RCCs in the studied period.

In the PCs from our study, mostly bacteria belonging to the skin and gut microbiota were found, which is consistent with previously published data [18, 20, 21, 22, 23]. The most common organism isolated from all PCs was *C. acnes*. It was isolated from 78.9% of all CP PCs, predominantly from anaerobic bottles (79.8%), with an average of 100.6 h of detection time (~4 days). The clinical significance of *C. acnes* is still debated; it is usually associated with minimal morbidity and mortality [24]. Although some studies report lack of proliferation and eventual bacterial death in PCs within days of incubation, *C. acnes* is an organism of clinical relevance in hospital settings often involved in infectious endocarditis and prosthetic infections [24, 25].

The anaerobic coagulase‐negative staphylococcus *Staphylococcus saccharolyticus* was found in 9.6% of all CP PCs. Neither *S. saccharolyticus* nor *C. acnes* proliferate during storage in PCs, yet both survive, and the possibility of transfusing biofilms has been documented as well [26]. Although rarely associated with infection, a transfusion reaction with *S. saccharolyticus* is possible, and its infectious potential should not be underestimated [27, 28].

Other bacteria identified from BCs at CITM were *S. aureus*, coagulase‐negative staphylococci (CoNS), *E. cloacae*, *E. coli*, *Serratia marcescens*, *Bacillus* spp. and *Streptococcus pyogenes*. Data show that all these microorganisms isolated from PCs are linked to transfusion‐associated sepsis with Gram‐negative organisms linked to a larger number of fatalities [9].

PCs are released as ‘negative to date’ but cannot be administered ≤12 h after inoculation for pooled products and ≤16 h for aphaeresis PCs, except in rare cases of critical platelet stock. Typically, PCs are administered after at least ≥20 h of incubation. All aerobic, more virulent bacterial species, including *E. coli*, *E. cloacae*, *S. aureus* and various CoNS, flagged positive within ≤20 h. Rapid detection of bottles containing pathogenic bacteria enables the recall of BCs potentially contaminated with isolates associated with higher morbidity and mortality rates before administration.

Statistical control of BCs continued after the implementation of a 100% screening of PCs. During this period, no cases of contamination were identified in any of the samples subjected to statistical QC.

Before implementation of LVDS, using the two‐step method for aphaeresis PCs, *S. aureus* was detected during the second step by performing a secondary culture and later confirmed in the retention sample. The initial sample remained negative. Although none of the components were transfused to a patient, this event highlighted the problem of false‐negative blood cultures due to early sampling, probably because of low initial inoculum numbers and thus no or insufficient presence of microorganisms in the inoculum. The negative status can also be the result of bottle under‐inoculation or an unsupportive culture bottle/medium.

Bacteria isolated from both CP and UP BCs were predominantly recovered from anaerobic bottles. Growth of bacterial pathogens, such as *E. coli*, *E. cloacae* and *S. aureus*, was observed not only in aerobic but also in anaerobic bottles, as these bacteria are also able to grow under anaerobic conditions. Growth of anaerobic bacteria, such as *C. acnes*, was also observed in both bottle types and, interestingly, sometimes only in BPA bottles. However, the detection time for *C. acnes* in aerobic bottles was notably longer. While some authors noted variable detection and a faster detection time of pathogenic bacteria in anaerobic bottles [29], such an effect was not noted in our study, nor in some other studies [20].

Although some studies debate a higher contamination rate among pooled products, certain studies note a similar contamination rate of single donor aphaeresis platelets and pooled platelets [29]. In this study, we report a total platelet product contamination incidence of 0.18% (0.14% for aphaeresis platelets and 0.20% for pooled platelets), which is similar to comparable studies [23, 30, 31].

The contamination rate of all PCs did not change significantly after the implementation of 100% screening of platelet products. However, a much larger absolute number of components was found to be contaminated with bacteria, since a much larger number of components has been screened, directly indicating a lower risk of TTI. Moreover, some microorganisms like *S. saccharolyticus* have been detected for the first time at CITM.

During the study period, FP results were also observed, which could be due to the device or bottles used, introduction of a new procedure, different interferences of the automated system, ambient temperature variations, as well as other numerous intrusions [11]. Studies showed a predominance of FPs in anaerobic bottles [11, 12]. A higher percentage of FP anaerobic BPN bottles was also noted at CITM in a previous study from 2012 [16]. In our study, we observed 2.4 times more FP aerobic bottles than anaerobic ones (91 vs. 38). Notably, 54 of all FP bottles have been recorded in 2023, with 94.4% positive aerobic bottles (51/54). Such a result could be linked to a specific bottle lot/series, temperature or device issue. An additional BacT/ALERT® Virtuo® has been implemented as of July 2023. In 2024, significantly fewer FP results have been recorded.

Beside bacterial screening, other measures can be implemented to reduce TTI, such as PRTs or rapid bacterial detection tests.

Both bacterial screening and PRTs require significant financial investments, more so on the PRT side, and careful planning and timing of processes, all of which is a big challenge for blood banks. PRTs reduce not only bacteria, but also viral and parasitic load. However, no PRT guarantees 100% pathogen removal, and reduction capacity differs for different technologies used [32]. There have been reports of TTIs with pathogen reduced BCs [33, 34]. Pathogen reduction can be performed on PCs and plasma, but not on RCCs. Through LVDS bacterial screening of whole blood derived PCs, entire blood donations from which the screened PCs were made are also indirectly screened and other contaminated components can be identified and possibly withdrawn if needed. Any additional processing of BCs affects their quality, so careful balancing of the component safety and quality must be considered when planning to introduce any new procedure. PRTs affect the quality of PCs mainly due to molecular damage and metabolic changes causing lower clinical efficacy of platelets [35] and LVDS bacterial screening causes higher average PC age at time of issue due to incubation and a slightly lower volume and number of platelets because of sampling.

At our institute, we validated one of the commercially available PRTs. The quality of PCs was seriously affected. Due to the quality issues, the shelf life of PCs could not be extended from 5 to 7 days as originally planned. The patients' response to PRT platelets was significantly lower compared to standard platelets (results not published, but consistent with some published studies) [35, 36]. These, and the financial load that comes with introducing PRTs, were the main reasons why we opted to implement 100% bacterial screening of PCs rather than PRT. A similar experience was described in the Netherlands [37]. The large number of samples tested over a long period, along with the comprehensive approach to bacterial testing of BCs enabled by the microbiology laboratory located at the institute, are the main strengths of this study. Comparing different sampling strategies provides valuable insights but is also a limiting factor in analysing trends in bacterial contamination over the study period.

In conclusion, while BC screening for bacteria along with other implemented measures has helped decrease the number of transfusion‐associated morbidity and mortality to an all‐time low, the risk remains. Even though most isolates were low pathogenic microorganisms, such as *C. acnes*, with a questionable clinical impact, transfusion of bacterially contaminated BCs presents an important health risk for transfused patients. Active monitoring of BCs, along with other measures, remains a critical step for ensuring transfusion safety and preventing TTIs.

## CONFLICT OF INTEREST STATEMENT

The authors declare no conflicts of interest.

## Data Availability

The data that support the findings of this study are available from the corresponding author upon reasonable request.
